# Lymph node metastasis after endoscopic submucosal dissection of a superficial esophageal adenocarcinoma arising from the ectopic gastric mucosa of the cervical esophagus: A case report

**DOI:** 10.1002/deo2.214

**Published:** 2023-02-21

**Authors:** Mamoru Ito, Akira Dobashi, Moe Komori, Shun Sugimura, Daisuke Aizawa, Keita Takahashi, Yuichiro Tanishima, Kazuki Sumiyama

**Affiliations:** ^1^ Department of Endoscopy The Jikei University School of Medicine Tokyo Japan; ^2^ Department of Gastroenterology and Hepatology The Jikei University School of Medicine Tokyo Japan; ^3^ Department of Pathology The Jikei University School of Medicine Tokyo Japan; ^4^ Department of Surgery The Jikei University School of Medicine Tokyo Japan

**Keywords:** case report, ectopic gastric mucosa, endoscopic submucosal dissection, esophageal adenocarcinoma, lymph node metastasis

## Abstract

Esophageal adenocarcinoma derived from the ectopic gastric mucosa of the cervical esophagus is very rare. Little is known about the efficacy of endoscopic treatment of these superficial lesions. Herein, we report the first case of lymph node metastasis after endoscopic submucosal dissection of a lesion with invasion into the muscularis mucosa. A 46‐year‐old man underwent esophagogastroduodenoscopy during a health checkup. Endoscopy revealed a 10‐mm‐sized nodular and a 5‐mm‐sized depressed lesion within the ectopic gastric mucosa of the cervical esophagus. The biopsy specimen confirmed the presence of adenocarcinoma. The entire ectopic gastric mucosa was resected by endoscopic submucosal dissection, and pathological examination showed invasion of the muscularis mucosa. A follow‐up computed tomography scan revealed lymph node metastasis 12 months post‐treatment. The patient underwent surgical mediastinal lymphadenectomy. The patient has been regularly followed up with a computed tomography scan and endoscopy for 2 years post‐surgery with no evidence of recurrence. Close follow‐up or additional treatment after endoscopic submucosal dissection should be considered and discussed with the patient if invasion into the muscularis mucosa is observed on pathological examination.

## INTRODUCTION

Ectopic gastric mucosa (EGM) is an area of gastric epithelium commonly situated on the cervical esophagus. Incomplete replacement of the early embryonic columnar epithelium causes this condition. EGM has a prevalence of 0.1%–10%^1^ and is frequently missed as the upper esophageal sphincter tightens the lumen of the cervical esophagus. The risk of neoplastic transformation is relatively low compared with that of Barrett's esophagus.^2^ Diagnosis with magnifying endoscopy and narrow‐band imaging is known to be effective in detecting early superficial carcinoma within the EGM.^3^

Early detected lesions can be treated by endoscopic resection rather than esophagectomy, which can cause severe complications such as leakage and stenosis at the anastomosis or vocal cord paralysis. Pech et al. reported the first case of endoscopic mucosal resection (EMR) for superficial esophageal adenocarcinoma arising from the EGM in 2001.^4^ Nonaka et al. reported the first case of endoscopic submucosal dissection (ESD) in 2013.^5^ ESD is advantageous over EMR in achieving en bloc resection for accurate histopathological assessment and staging regardless of lesion size. Even so, the efficacy and long‐term prognosis of ESD for EGM lesions remain unclear. Herein, we report a case of lymph node metastasis salvaged by surgical mediastinal lymphadenectomy after ESD of an esophageal adenocarcinoma on EGM confined to the muscular mucosa.

## CASE

A 46‐year‐old male with a history of type 2 diabetes mellitus and hiatal hernia underwent esophagogastroduodenoscopy (EGD). He had a 20‐pack‐year history of cigarette smoking and drank occasionally with flushing. EGD found a 10‐mm polypoid lesion on the distal edge of the EGM in the cervical esophagus, and a biopsy of the lesion revealed adenocarcinoma. The patient was referred to our hospital for further treatment.

**FIGURE 1:**
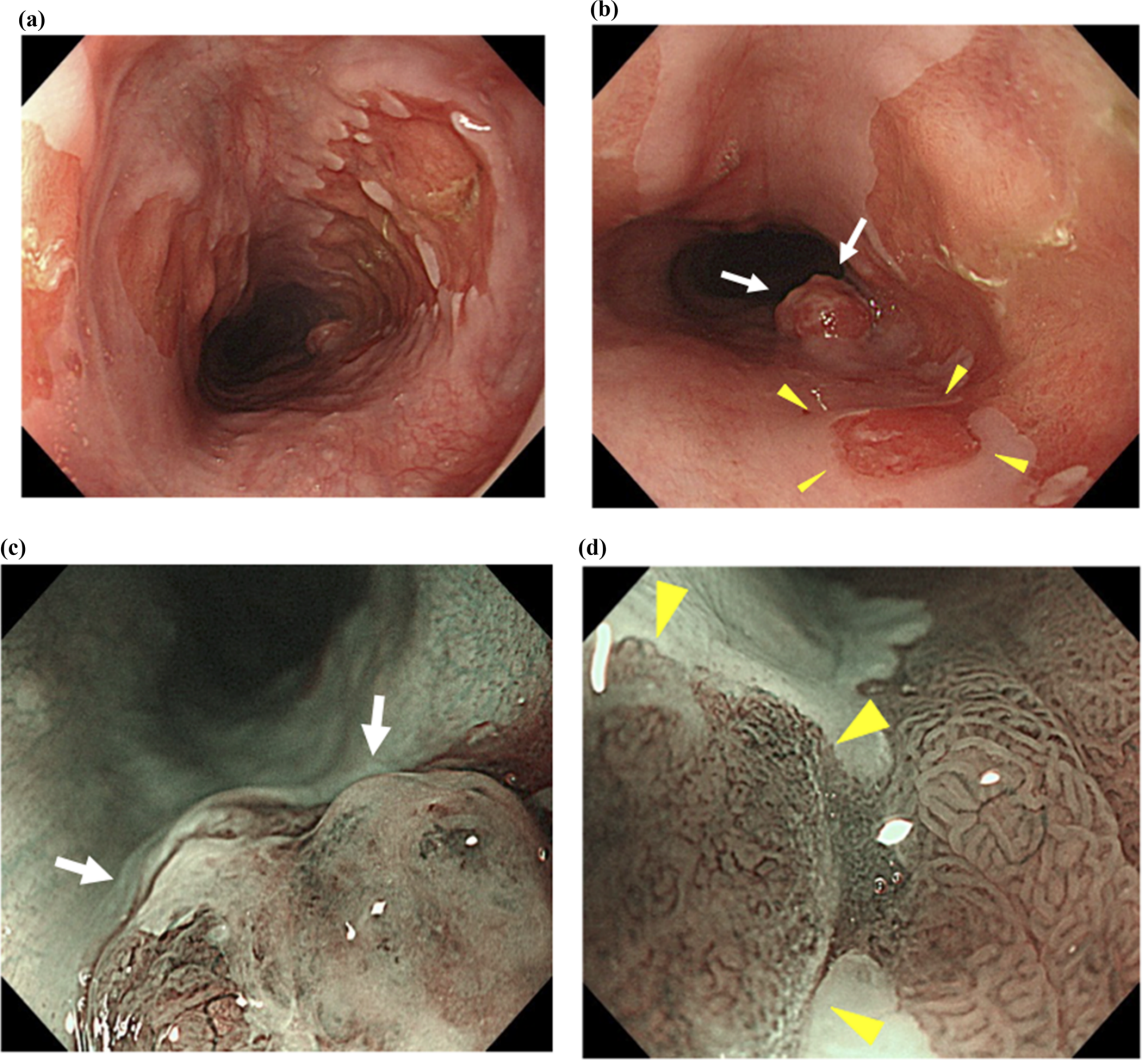
(a) White‐light imaging revealed ectopic gastric mucosa in the cervical esophagus. (b) A nodular lesion (white arrows) and a reddish depressed lesion (yellow arrowheads) were found on the ectopic gastric mucosa's anal edge and left edge. (c) Magnifying narrow‐band imaging of the nodular lesion (white arrows) showing irregular microvascular and microsurface patterns. (d) Magnifying narrow‐band imaging of the depressed lesion (yellow arrowheads) showing irregular microvascular and microsurface patterns.

EGD revealed a large EGM 40 mm in size at the cervical esophagus, 16 cm from the incisor. A 10‐mm reddish polypoid 0‐Is lesion arose from the distal edge of the EGM (Figure 1a,b). Narrow‐band imaging combined with magnifying endoscopy revealed irregular microvascular and microsurface patterns (Figure 1c). In addition, a 5‐mm reddish 0‐IIc lesion was found at the left edge of the EGM approximately 1 cm orally also with irregular microvascular and microsurface patterns (Figure 1b,d). A biopsy specimen of this lesion revealed adenocarcinoma. Normal esophageal mucosa intervened in both lesions (Figure S1). These findings suggested the presence of multiple cancers or subepithelial extensions under the squamous epithelium. Endoscopic ultrasound with a 20 MHz mini‐probe for the 0‐Is lesion showed seven layers of the esophageal wall and depicted a low‐echoic mass in the second and third layers.

The treatment options included surgical resection or endoscopic resection; the patient consented to the latter. The entire EGM with a sufficient margin including normal squamous epithelium was excised using en bloc ESD considering the subepithelial invasion under general anesthesia (Figure S2). No complications such as post‐ESD bleeding or perforation were observed.

**FIGURE 2:**
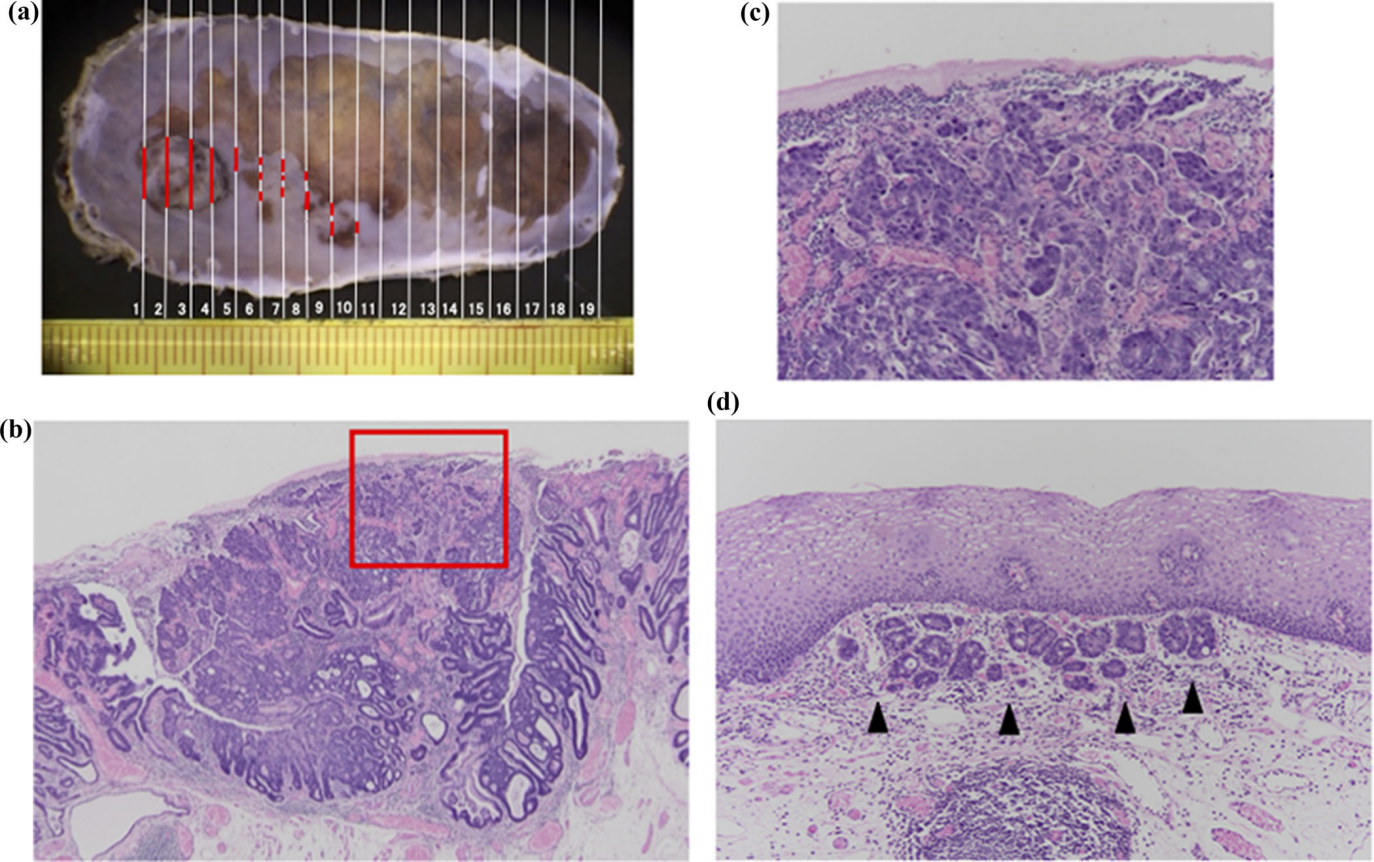
(a) Gross macroscopic image of the resected specimen, with red lines mapping carcinoma. Subepithelial invasion (#5–8) was seen between the nodular lesion (#1–4) and the depressed lesion (#8–10). (b) Microscopic pathological image with hematoxylin–eosin (staining of the nodular lesion shows well to moderately differentiated adenocarcinoma (×4). Poorly differentiated components are seen near the surface (red box). (c) Magnified poorly differentiated components (×10). The image is corresponding to the red box in Figure 2(b). (d) Microscopic pathological image with hematoxylin–eosin staining of subepithelial invasion (black arrows; ×10).

Pathologica examination showed Ce, 28 × 8 mm, 0‐IIc + Is, well‐to‐moderately differentiated tubular adenocarcinoma, pT1a‐MM, ly0, v0, pHM0 pVM0, and pR0.Notably, the lesion extended horizontally beneath the healthy squamous epithelium from the nodule to the depressed lesion (Figure 2a,d). Well to moderately differentiated cells comprised the nodular lesion with partial poorly differentiated cells at the surface (Figure 2b,c). Meanwhile, well‐differentiated cells constituted the subepithelial invasion and depressed lesion. Desmin staining revealed the invasion of the muscular mucosa (Figure S3). Immunohistochemical studies revealed CD10 positive with focal positivity of MUC5AC, MUC6, and MUC2, indicating gastric‐intestinal mixed type with the dominant intestinal component (Figure S4a–d).

**FIGURE 3:**
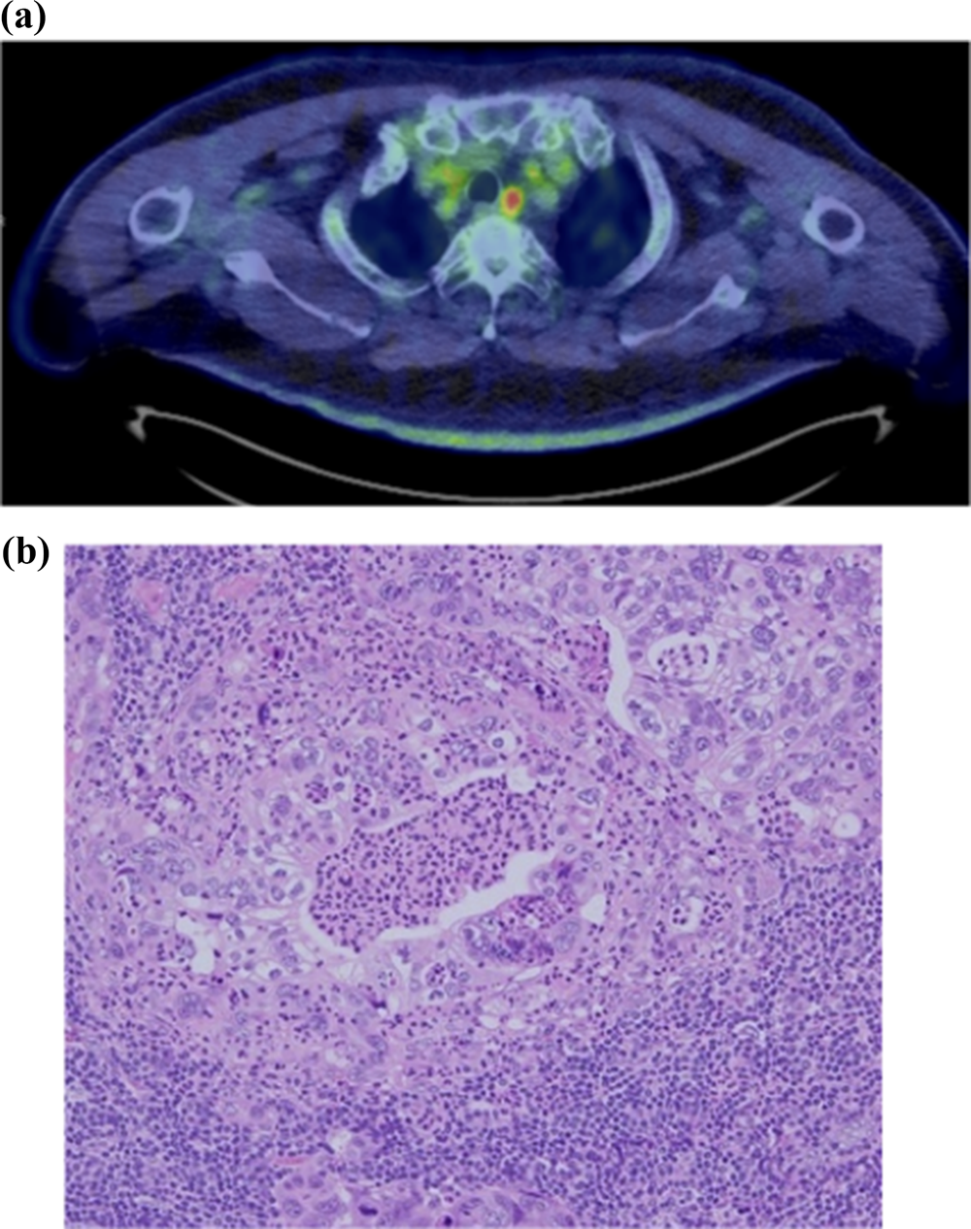
Lymph node metastasis was found 12 months after the endoscopic resection (a) Positron emission tomography‐computed tomography showed high fluorodeoxyglucose uptake at the 106‐rec lymph node. (b) Histopathological image with hematoxylin and eosin staining showed adenocarcinoma similar to the endoscopically resected esophageal lesion.

The possibility of metastasis was explained and the patient preferred close follow‐up with computed tomography (CT) every 6 months without further treatment. Metastasis to the lymph node was diagnosed 12 months later when the CT showed an enlarged 106‐rec lymph node and positron emission tomography‐CT imaging demonstrated high fluorodeoxyglucose uptake (Figure 3a). Surgical mediastinal lymphadenectomy was performed and pathology of the resected specimen showed adenocarcinoma with a histological appearance similar to that of esophageal adenocarcinoma (Figure 3b). The patient was followed up with EGD every 6 months and CT every 4 months, and no recurrence was noted 24 months postoperatively.

## DISCUSSION

This is the first report of mucosal adenocarcinoma arising from the EGM in the cervical esophagus, which caused lymph node metastasis after endoscopic resection. We summarize past reports of endoscopic treatment performed for adenocarcinoma of the cervical esophagus derived from EGM in Table 1. ESD has been the treatment of choice over EMR in recent years including for lesions smaller than 10 mm. Long‐term follow‐up data are limited although curative resection is achieved in most cases. Kitasaki et al. reported local recurrence after ESD of cervical esophageal adenocarcinoma confined to the muscular mucosa where subtotal resection of EGM was performed, which implies metachronous cancer in the residual EGM^6^ or subepithelial invasion under the normal mucosa like Barrett's adenocarcinoma. We completely resected the entire EMG with a sufficient margin including the normal mucosa considering metachronous cancer and subepithelial invasion, and no local recurrence was observed in our case.

**TABLE 1:** Reported cases of adenocarcinoma originating in ectopic gastric mucosa treated by endoscopy

Case	Author	Year	Age (years)	Sex	Smoking	Size (mm)	Depth	Initial treatment	Additional treatment	Follow‐up period
1	Pech et al.^4^	2001	77	M	NA	NA	m	EMR	(‐)	12 months
2	Hirayama et al.^10^	2003	77	M	NA	21	m	EMR	(‐)	31 months
3	Yoshida et al.^11^	2009	79	M	NA	4	m	EMR	(‐)	2 years
4	Nonaka et al.^5^	2013	74	M	NA	7	Mm?	ESD	(‐)	NA
5	Möschler et al.^12^	2014	83	M	NA	NA	M	EMR	(‐)	NA
6	Yasar et al.^13^	2014	52	F	(‐)	7	m	ESD	(‐)	31 months
7	Nomura et al.^14^	2015	62	M	(+)	49	mm	ESD	(‐)	12 months
8	Probst et al.^15^	2015	62	M	NA	7	m	ESD	(‐)	NA
9	Hudspeth et al.^16^	2016	77	M	(+)	15	m	EMR	(‐)	1 year
10	Gushima et al.^17^	2017	65	F	(–)	16	sm	ESD	Chemotherapy	40 months
11	Tanaka et al.^18^	2018	69	M	NA	10	m	ESD	(‐)	NA
12	Tanaka et al.^19^	2019	72	M	NA	8	mm	ESD	(‐)	24 months
13	Oono et al.^20^	2019	58	M	()	32	mm	ESD	(‐)	NA
14	Ohki et al.^21^	2021	59	M	()	44	mm	ESD	(‐)	1 year
15	Kitasaki et al.^6^	2022	52	M	(+)	20	mm	ESD	ESD twice + surgery	40 months
16	Our case	2022	46	M	(+)	28	mm	ESD	Surgery	39 months

Abbreviations: EMR, endoscopic mucosal resection; ESD, endoscopic submucosal dissection; F, female; M, male; NA, not available.

References 10–21 are listed in the Supporting Information.

There is a low rate of metastasis in a muscular mucosal invasion of the adenocarcinoma at the esophagogastric junction so‐called Barrett's adenocarcinoma. The ESD/EMR guidelines for esophageal cancer report lymph node metastasis rates of 0% (95% confidence interval 0–2.5) in 105 cases of pathologically diagnosed esophageal adenocarcinoma invading the deep muscular mucosa with negative vascular invasion after surgical resection.^7^ This study was limited to lesions with well‐differentiated adenocarcinomas, and those with poorly differentiated components were excluded. In a multicenter retrospective study in a Japanese population, 72 patients (31 surgically resected and 41 endoscopically resected) out of 458 patients with esophageal adenocarcinoma or esophagogastric adenocarcinoma were identified with lymph node metastasis.^8^ Multivariate analysis revealed the following as independent risk factors for metastasis; lymphovascular involvement, a poorly differentiated component, and a lesion size >30 mm. Poorly differentiated components were observed in our case which might have contributed to the lymph node metastasis. It remains to be seen if the same risk factors can be applied in the case of EGM‐derived adenocarcinomas and if more evidence is needed.

In our case, horizontal invasion beneath the healthy squamous epithelium was observed, which is congruent with findings in Barrett's adenocarcinoma.^9^ Endoscopists should be aware of this possibility in the pre‐treatment observation of EGM‐derived adenocarcinoma and we recommend resection of the entire EGM.

To our knowledge, this is the first report of lymph node metastasis after ESD of a cervical esophageal adenocarcinoma derived from an EGM. Our case highlights the importance of removing the entire EGM with normal squamous epithelium and the possibility of post‐ESD lymph node metastasis. Lesions invading the muscularis mucosa with poorly differentiated components should be closely followed‐up with CT considering lymph node recurrence.

## CONFLICT OF INTEREST STATEMENT

K.S. is a Deputy Editor‐in‐Chief of DEN Open. The rest of the authors declare no conflict of interest.

## Supporting information


**Figure S1**: Normal epithelium between nodular lesion (white arrows) and depressed lesion (yellow arrowheads).

**Figure S2**: Gross macroscopic image of the resected specimen with nodular (white arrows) and depressed (yellow arrowheads) lesions. The entire EGM is resected along with the lesion.

**Figure S3**: Desmin staining reveals aberrant muscular mucosa (×4).

**Figure S4a**: Immunohistochemical staining of abnormal glandular structure showing focal MUC5AC positivity (×4).

**Figure S4b**: Focal MUC6 positivity (×4).

**Figure S4c**: Focal MUC2 positivity (×4).

**Figure S4d**: (d) CD10 positivity (×4).

References
