# Supplementary figures and images for: Lymph node metastasis after endoscopic submucosal dissection of a superficial esophageal adenocarcinoma arising from the ectopic gastric mucosa of the cervical esophagus: A case report
Source: DEN Open. 2023 Feb 21;3(1):e214. doi: 10.1002/deo2.214 (PMC9942940; doi:10.1002/deo2.214)

Supporting Information

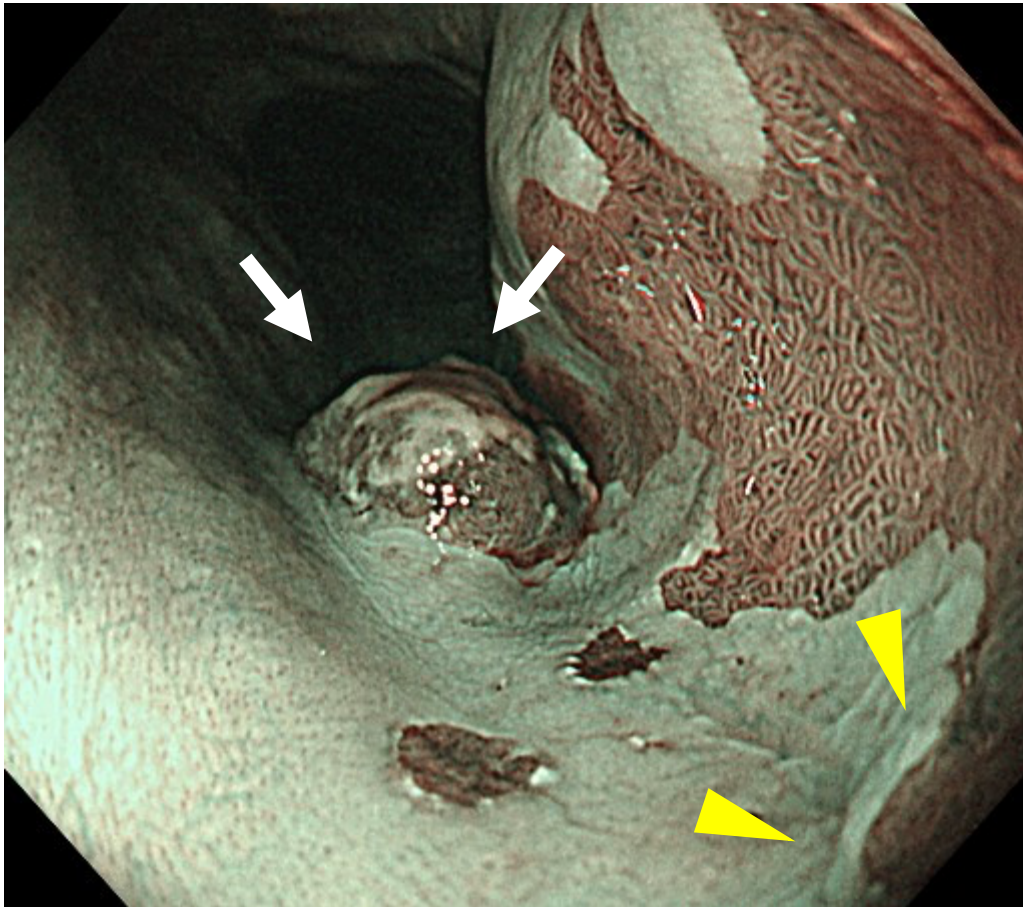

Figure S1.

Supplement: Supplementary file 1 — Figure S1: Normal epithelium between nodular lesion (white arrows) and depressed lesion (yellow arrowheads). [file DEO2-3-e214-s002.pdf]

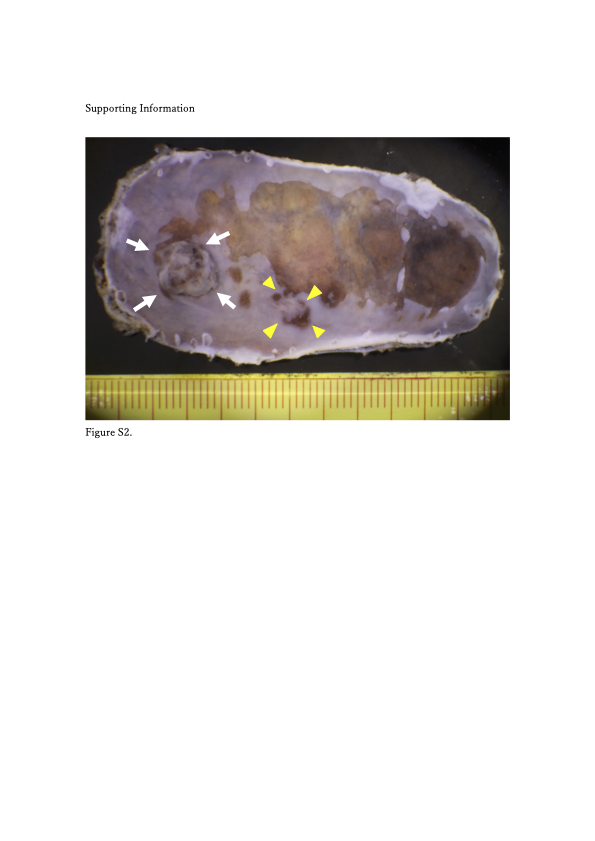

Supplement: Supplementary file 2 — Figure S2: Gross macroscopic image of the resected specimen with nodular (white arrows) and depressed (yellow arrowheads) lesions. The entire EGM is resected along with the lesion. [file DEO2-3-e214-s001.tiff]

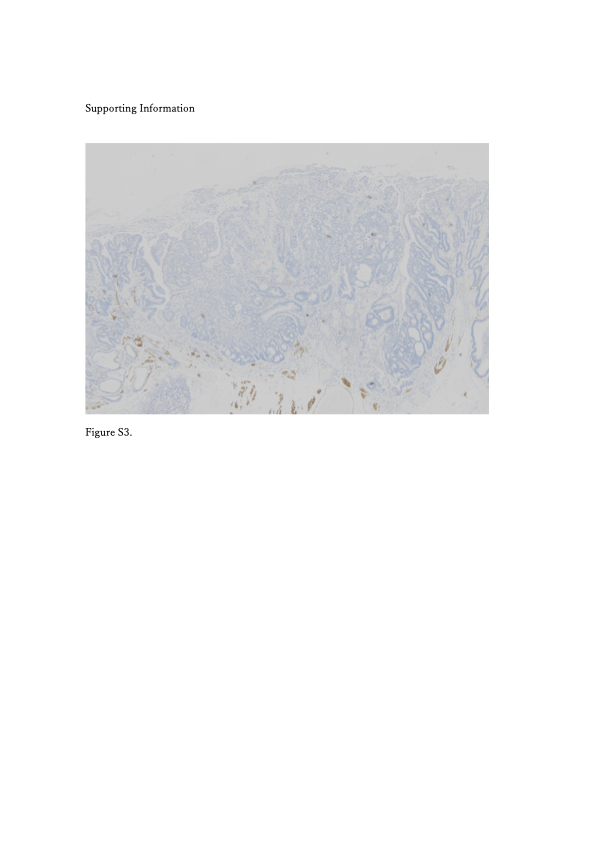

Supplement: Supplementary file 3 — Figure S3: Desmin staining reveals aberrant muscular mucosa (×4). [file DEO2-3-e214-s005.tiff]

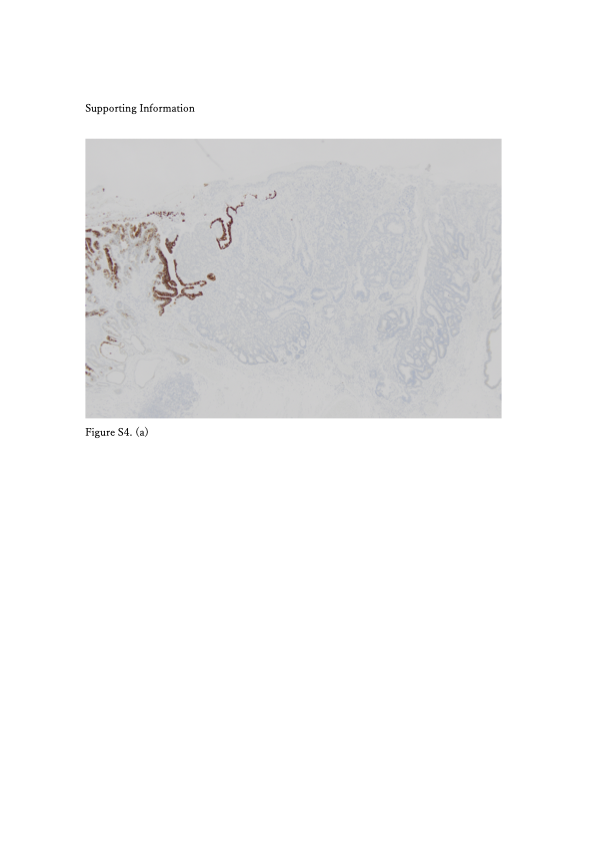

Supplement: Supplementary file 4 — Figure S4a: Immunohistochemical staining of abnormal glandular structure showing focal MUC5AC positivity (×4). [file DEO2-3-e214-s008.tiff]

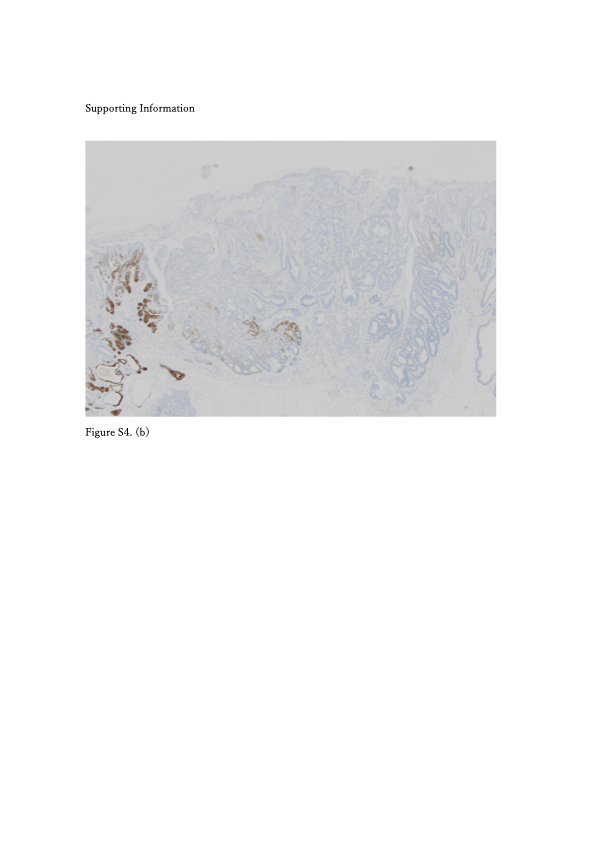

Supplement: Supplementary file 5 — Figure S4b: Focal MUC6 positivity (×4). [file DEO2-3-e214-s007.tiff]

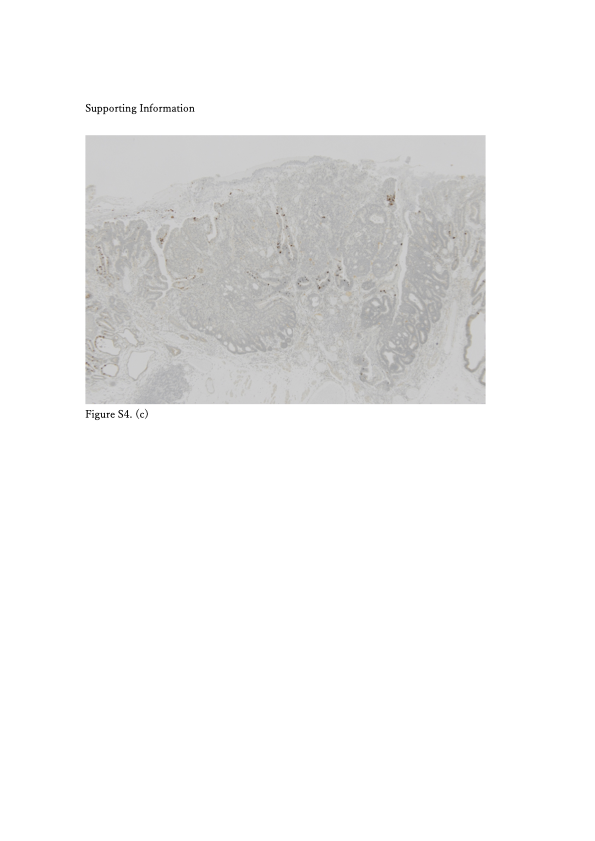

Supplement: Supplementary file 6 — Figure S4c: Focal MUC2 positivity (×4). [file DEO2-3-e214-s004.tiff]

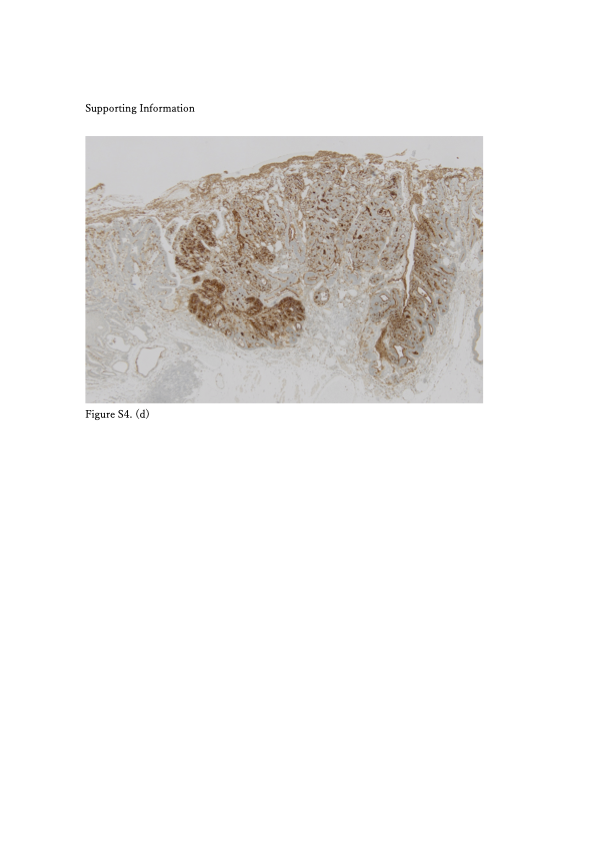

Supplement: Supplementary file 7 — Figure S4d: (d) CD10 positivity (×4). [file DEO2-3-e214-s006.tiff]
