# Supplementary material for: Lymph node metastasis after endoscopic submucosal dissection of a superficial esophageal adenocarcinoma arising from the ectopic gastric mucosa of the cervical esophagus: A case report
Source: DEN Open. 2023 Feb 21;3(1):e214. doi: 10.1002/deo2.214 (PMC9942940; doi:10.1002/deo2.214)
Supplement: Supplementary file 8 — References [file DEO2-3-e214-s003.docx]

Supporting Information

References

10. Hirayama N, Arima M, Miyazaki S, et al. Endoscopic mucosal resection of adenocarcinoma arising in ectopic gastric mucosa in the cervical esophagus: case report. *Gastrointest Endosc* 2003; 57(2): 263-6.

11. Yoshida T, Shimizu Y, Kato M. Image of the month. Use of magnifying endoscopy to identify early esophageal adenocarcinoma in ectopic gastric mucosa of the cervical esophagus. *Clin Gastroenterol Hepatol* 2010; 8(9): 91-3.

12. Möschler O, Vieth M, Muller MK. Endoscopic resection of an adenocarcinoma occurring in ectopic gastric mucosa within the proximal esophagus. *Endoscopy* 2014; 46 Suppl 1: 24-5.

13. Yasar B, Tarcin O, Benek D, et al. Intramucosal adenocarcinoma arising from ectopic gastric mucosa in the upper esophagus treated successfully with endoscopic mucosal resection. *J Gastrointest Cancer* 2014; 45 Suppl 1: 201-4.

14. Nomura K, Iizuka T, Inoshita N, et al. Adenocarcinoma of the cervical esophagus arising from ectopic gastric mucosa: report of two cases and review of the literature. *Clin J Gastroenterol* 2015; 8(6): 367-76.

15. Probst A, Schaller T, Messmann H. Adenocarcinoma arising from ectopic gastric mucosa in an esophageal inlet patch: treatment by endoscopic submucosal dissection. *Endoscopy*. 2015; 47 Suppl 1: 337-8.

16. Hudspeth VR, Smith DS, Pacicco T, et al. Successful endoscopic resection of adenocarcinoma arising in an esophageal inlet patch. *Dis Esophagus* 2016; 29(7): 880-2.

17. Gushima R, Narita R, Shono T, et al. Esophageal adenocarcinoma with enteroblastic differentiation arising in ectopic gastric mucosa in the cervical esophagus: a case report and literature review. *J Gastrointestin Liver Dis* 2017; 26(2): 193-7.

18. Tanaka K, Fujiwara M, Toyoda H. An Unlikely Lesion to Be Identified in the Cervical Esophagus. *Gastroenterology* 2018; 155(3): 610-2.

19. Tanaka K, Yamada R, Tsuboi J, et al. A small adenocarcinoma in the cervical esophagus. *VideoGIE* 2019; 4(3): 111-3.

20. Oono Y, Kensuke S, Yoda Y, et al. Cervical esophageal adenocarcinoma arising from heterotopic gastric mucosa, treated with endoscopic submucosal dissection. *Endoscopy* 2019; 51(2): 28-9.

21. Ohki D, Tsuji Y, Yamazawa S, et al. Gastrointestinal: Esophageal adenocarcinoma arising from circumferential ectopic gastric mucosa: A case report. *J Gastroenterol Hepatol* 2022; 37(1): 47.
